# Tracheostomy and Ventilator-Associated Pneumonia in Mechanically Ventilated ICU Patients: A Retrospective Matched Cohort Study

**DOI:** 10.3390/jcm15124811

**Published:** 2026-06-21

**Authors:** Marie Nicoline Ordaz-Kücks, Iván Alejandro Arteaga-Martínez, Hugo Alfredo Funes-González, Fernando Martín Guerra-Infante, Roberto Montes-de-Oca-Jiménez, Martha Elba Ruiz-Riva-Palacio, Javier Morales-Fabian, Enrique Rojano-Lastra, Heberto Hernández-Miranda, José Carlos Aguilar-Carrasco, Gabriel Arteaga-Troncoso

**Affiliations:** 1 Programa Académico de la Especialidad en Epidemiología, Hospital General “Dra. Matilde Petra Montoya Lafragua”, Ciudad de México 13260, Mexico; maniorku@gmail.com (M.N.O.-K.); coxpeckcuatro@gmail.com (H.A.F.-G.); salmonelladt104@hotmail.com (J.M.-F.); enrique.rojano@issste.gob.mx (E.R.-L.); heberto1973@yahoo.com.mx (H.H.-M.); 2 Escuela Superior de Medicina, Instituto Politécnico Nacional, Ciudad de México 11340, Mexico; iarteagam2000@alumno.ipn.mx; 3 Departamento de Fisiología y Desarrollo Celular, Instituto Nacional de Perinatología “Isidro Espinosa de los Reyes”, Ciudad de México 11000, Mexico; fernando.guerra@inper.gob.mx; 4 Facultad de Medicina Veterinaria y Zootecnia, Universidad Autónoma del Estado de México, Toluca 56200, Estado de México, Mexico; rmontesdeocaj@uaemex.mx; 5 Plantel Sor Juana Inés de la Cruz, Universidad Autónoma del Estado de México, Amecameca 56900, Estado de México, Mexico; meruizr@uaemex.mx; 6 Laboratorio de Farmacología Experimental, Instituto Nacional de Perinatología “Isidro Espinosa de los Reyes”, Ciudad de México 11000, Mexico

**Keywords:** ventilator-associated pneumonia, tracheostomy, mechanical ventilation, intensive care units, retrospective matched cohort study, antimicrobial resistance, Gram-negative pathogens, critical care

## Abstract

**Background/Objectives**: Ventilator-associated pneumonia (VAP) remains a major complication in patients requiring prolonged mechanical ventilation. The effect of tracheostomy on VAP risk remains controversial, particularly when differences in duration of mechanical ventilation are considered. This study evaluated the association between tracheostomy, VAP occurrence, and clinical outcomes in mechanically ventilated ICU patients. **Methods**: We conducted a retrospective matched exposed–unexposed cohort study in a tertiary-care ICU in Mexico City. Patients undergoing tracheostomy were compared with an age- and sex-matched subcohort of intubated patients receiving invasive mechanical ventilation for ≥48 h. VAP incidence was assessed using cumulative incidence, incidence density, and multivariable generalized linear models. **Results**: A total of 218 patients were included (55 tracheostomized and 163 intubated). VAP incidence density was similar between groups (31.5 vs. 30.3 per 1000 ventilator-days; RR 1.04, 95% CI 0.7–1.7), whereas cumulative incidence was higher among tracheostomized patients (61.8% vs. 22.7%; RR 2.7, 95% CI 1.9–3.9). Broad-spectrum antibiotics, mechanical ventilation ≥ 5 days, chronic pulmonary disease, and ICU stay remained associated with VAP occurrence in an exploratory multivariable model. Gram-negative microorganisms predominated, and antimicrobial resistance was more frequent among tracheostomized patients. **Conclusions**: Tracheostomy was associated with higher cumulative incidence of VAP, but a similar incidence density compared with endotracheal intubation. The crude association between tracheostomy and VAP disappeared after adjustment for confounding factors, suggesting that prolonged mechanical ventilation and ICU exposure are more important determinants of VAP risk than tracheostomy itself.

## 1. Introduction

Nosocomial infections, also referred to as healthcare-associated infections (HAIs), originate within the hospital setting and remain a major cause of morbidity and mortality, especially in low- and middle-income countries [1]. Recent evidence highlights that hospital-acquired pneumonia (HAP) and ventilator-associated pneumonia (VAP) represent a continuum of nosocomial lower respiratory tract infections with overlapping pathophysiological mechanisms, microbiological profiles, and diagnostic challenges, reinforcing the need for integrated surveillance and prevention strategies [2].

Data from the International Nosocomial Infection Control Consortium (INICC) reveal the burden of HAIs across the World Health Organization regions, including 45 countries from Latin America, Europe, the Eastern Mediterranean, Southeast Asia, and the Western Pacific, between 2012 and 2017. This analysis identified 16,099 VAP cases, with a pooled mean rate of 14.1 per 1000 ventilator-days in intensive care units [3]. VAP is typically defined as a nosocomial infection occurring after 48 h of mechanical ventilation and is commonly caused by infectious agents such as *Pseudomonas aeruginosa*, *Escherichia coli*, *Klebsiella pneumoniae*, *Acinetobacter* sp., *Streptococcus pneumoniae*, and *Staphylococcus aureus*, among others [4,5]. Sequential evaluation of tracheostomized patients, particularly those requiring prolonged mechanical ventilation (PMV), is essential for the early detection of VAP.

A tracheostomy is primarily undertaken to facilitate weaning from mechanical ventilation, ensure airway protection, and mitigate complications associated with prolonged endotracheal intubation [6]. Nevertheless, uncertainty persists regarding whether an early or late tracheostomy confers superior clinical benefits, particularly in relation to the risk of VAP when accounting for time-dependent exposure and competing clinical outcomes [7]. Despite advances in infection prevention and control strategies, VAP continues to be a major cause of morbidity and mortality among critically ill patients. Its occurrence is associated with a prolonged hospital stay, delayed extubation, increased utilization of healthcare resources, and the emergence of antimicrobial resistance factors, which have consistently been emphasized in recent comprehensive reviews of HAP/VAP [2,8]. Optimizing airway management strategies to improve patient safety and reduce the incidence of VAP remains a key priority in the prevention of HAIs.

Several studies have shown that tracheostomy may influence the duration of mechanical ventilation in critically ill patients, including those with acute respiratory distress syndrome (ARDS), COVID-19, and sepsis [9,10]. It has also been suggested that tracheostomy could reduce the risk of VAP by facilitating improved oral hygiene and decreasing oropharyngeal colonization by potentially pathogenic microorganisms [11]. However, the available evidence remains inconsistent across heterogeneous ICU populations and real-world clinical settings. While some observational studies and meta-analyses suggest that earlier tracheostomy may reduce the incidence of VAP and duration of mechanical ventilation, others have reported no significant association [12,13,14]. These discrepancies likely reflect differences in study design, patient populations, tracheostomy timing and methods used to account for duration of mechanical ventilation. Addressing this gap is essential for generating clinically meaningful evidence that can inform decision-making and optimize patient outcomes in critical care.

## 2. Materials and Methods

### 2.1. Study Design and Setting

A retrospective exposed–unexposed cohort study was conducted as part of the academic program at the Hospital General “Dra. Matilde Petra Montoya Lafragua”, Instituto de Seguridad y Servicios Sociales de los Trabajadores del Estado (ISSSTE), located in Mexico City, Mexico. The study evaluated the occurrence of VAP, incidence density, antimicrobial resistance patterns, ICU length of stay, and mortality among mechanically ventilated patients.

### 2.2. Ethical Considerations

This study was conducted in accordance with the Strengthening the Reporting of Observational Studies in Epidemiology (STROBE) guidelines. Ethical principles outlined in the Declaration of Helsinki and applicable national regulations for biomedical research and personal data protection were followed. All data were anonymized and handled confidentially. The study protocol was approved by the Institutional Research Ethics Committee (approval number: PR066/18), and the requirement for informed consent was waived due to the retrospective and anonymized nature of the data.

### 2.3. Study Population and Eligibility Criteria

**Figure 1:**
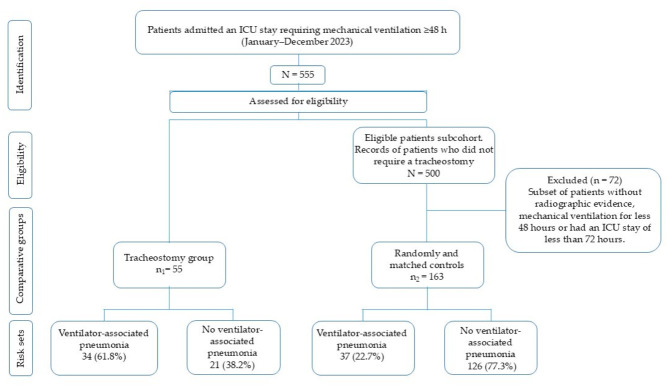
Flowchart of study design and patient selection. Two hundred and eighteen patients requiring invasive mechanical ventilation for ≥48 h were included. Patients were classified according to airway management into a tracheostomy group (cases) and a subcohort of randomly selected, age- and sex- matched controls without a tracheostomy. Patients were followed during hospitalization for the development of ventilator-associated pneumonia (VAP). A retrospective matched cohort study was implemented to optimize statistical efficiency. Outcomes were analyzed using cumulative incidence, incidence density, and logistic regression.

All patients admitted to the intensive care unit (ICU) between January and December 2023 who required invasive mechanical ventilation for more than 48 h were considered eligible (Figure 1). The primary outcome was the occurrence of VAP during hospitalization. Secondary outcomes included incidence density, antimicrobial resistance patterns, ICU length of stay, hospital length of stay, and mortality.

Cases were defined as patients who underwent a tracheostomy based on established clinical indications, including upper airway obstruction or persistent respiratory failure requiring prolonged mechanical ventilation. The decision to perform tracheostomy reflected clinical severity and the inability to sustain adequate spontaneous ventilation. Patients were prospectively monitored during hospitalization for the development of VAP. Diagnosis was established in mechanically ventilated patients presenting with (i) new or progressive pulmonary infiltrates on chest radiography; (ii) fever (>38 °C or <36 °C); (iii) leukocytosis or leukopenia; and (iv) at least one of worsening gas exchange, purulent respiratory secretions, or positive culture from respiratory specimens [13]. Patient records were excluded if they met any of the following criteria: (i) absence of radiographic data; (ii) duration of mechanical ventilation < 48 h; or (iii) ICU stay < 72 h. Additionally, records of VAP patients transferred from or referred to other institutions, as well as those diagnosed with non-ventilator-associated pneumonia, were excluded from the analysis.

In patients with pre-existing pulmonary or cardiac disease, the presence of infiltrates on two consecutive chest radiographs was required for diagnosis. Extubation failure was defined as the need for reintubation within 48–72 h after the removal of ventilator support due to the inability to maintain adequate spontaneous breathing.

### 2.4. Matched Cohort Sampling Strategy and Sample Size Calculation

The initial population included 555 patients, of whom 55 (9.9%) underwent a tracheostomy (cases), and 500 (90.1%) records of patients on mechanical ventilation were analyzed. To select the records of patients who served as the control group, the subsample size was calculated based on the following equation:

$$n_{2}=\frac{Z_{\alpha /2}^{2}\left(p_{1}q_{1}+kp_{2}q_{2}\right)}{k\delta^{2}}$$

Total population without a tracheostomy (N = 500);

Number of cases (*n*_1_ = 55);

Ratio N/*n*_1_ (*k* = 9.1);

Expected VAP incidence in tracheostomized patients (*p*_1_ = 0.618);

Expected incidence in intubate patients (*p*_2_ = 0.227);

*δ*^2^ = 0.01;

The minimum required control simple size was estimated at *n*_2_ = 158.

To reduce selection bias and increase statistical power, records of patients were randomly selected and matched by age and sex to cases, resulting in a final control group of 163 patients [15]. The post hoc statistical power (1 − β) for comparing between groups was 0.99, calculated using G*Power software (version 3.1.9.7).

### 2.5. Clinical Management

Both groups were managed by the same multidisciplinary team, including physicians, nurses, and respiratory therapists, under standardized institutional protocols. Researchers were not involved in direct patient care. This approach was implemented to minimize variability in clinical management and reduce potential confounding related to treatment differences.

### 2.6. Antimicrobial Susceptibility Testing

In cases of clinical suspicion of VAP, antimicrobial susceptibility testing (AST) was performed on isolates obtained from bronchial aspirate specimens against a panel of 41 antimicrobial agents. Bacterial identification and AST were conducted using the automated bioMérieux mini–API system (bioMérieux, Marcy-I’Étoile, France). Separate antibiograms were generated for Gram-negative and Gram-positive bacteria. Minimum inhibitory concentration (MIC) values were interpreted according to the criteria established by the Clinical and Laboratory Standards Institute [16]. Isolates resistant to more than three different classes of antimicrobials were classified as multidrug-resistant (MDR) according to international consensus definitions [17]. The multiple antibiotic resistance (MAR) index for each isolate was calculated as the ratio of the number of antimicrobials to which the isolate was resistant to the total number of antimicrobials tested [18].

### 2.7. Statistical Analysis

This study was conducted and reported in accordance with the STROBE statement. Continuous variables are presented as means ± standard deviations (SDs) or medians (interquartile range, IQR), depending on the data distribution. Categorical variables are expressed as frequencies and percentages. A two-tailed *p*-value of <0.05 was considered statistically significant. Normality was assessed using the Kolmogorov–Smirnov test. Comparisons between continuous variables were performed using Student’s *t*-test or the Mann–Whitney U test, as appropriate. Categorical variables were compared using Fisher’s exact test. Correlations were evaluated using Spearman’s rank correlation coefficient.

The risk of VAP was estimated using the cumulative incidence (proportion of patients who developed VAP during follow-up) and incidence density (number of new VAP cases divided by total ventilator-days, multiplied by 1000).

Univariable logistic regression analysis was conducted to identify factors associated with VAP. Continuous variables were dichotomized for analysis. The variables assessed included age, sex, body mass index (BMI), comorbidities, requirement for a tracheostomy, patient positioning (decubitus), use of sedation, duration of invasive mechanical ventilation, length of hospital stay, and mortality. The results are reported as regression coefficients, odds ratios (ORs), and 95% confidence intervals (95% CIs).

Variables with statistical significance in the univariable analysis were included in the generalized linear model, which was adjusted for age and gender. Adjusted effect estimates were obtained using a generalized linear model with binomial distribution and a complementary log–log link function. Effect estimates obtained from the complementary log–log model is presented as adjusted relative risk (aRR) with 95% confidence intervals (CIs), for consistency and interpretability. Model construction was as follows: (i) variables with statistical significance were initially included; (ii) each variable was evaluated using the Wald test (*p* < 0.05), as well as the Akaike Information Criterion (AIC) and Bayesian Information Criterion (BIC); (iii) variables that lost statistical significance after adjustment were manually removed; and (iv) the process was repeated until a stable final model was achieved. All statistical analyses were performed using IBM SPSS Statistics version 25 (IBM Corp., Armonk, NY, USA).

## 3. Results

### 3.1. Study Population and Baseline Characteristics

During the study period, 55 patients underwent a tracheostomy between days 5 and 29 after endotracheal intubation (median: 18 days; interquartile range [IQR]: 14–24). The comparative subcohort consisted of 163 patients randomly selected from those who underwent endotracheal intubation during the same period (Table 1). Of the 500 initially eligible records, 72 (14.4%) were excluded due to incomplete data related to VAP, including missing radiographic studies, duration of mechanical ventilation < 48 h, or ICU stay < 72 h.

### 3.2. Incidence of Ventilator-Associated Pneumonia During Follow-Up

To assess differences in VAP risk, we estimated the incidence density per 1000 ventilator-days and cumulative incidence and calculated IRRs and RRs. Incidence density did not differ between groups (31.5 vs. 30.3 cases per 1000 ventilator-days; IRR: 1.04, 95% CI: 0.7–1.7). However, cumulative incidence was significantly higher among tracheostomized patients (61.8%, 95% CI: 49.0–74.6) than intubated patients (22.7%, 95% CI: 16.3–29.1), yielding an RR of 2.7 (95% CI: 1.9–3.9). These findings indicate that, despite similar incidence rates over time, tracheostomized patients may have a higher overall probability of developing VAP during the follow-up period.

### 3.3. Clinical Outcomes and Mechanical Ventilation

Successful removal of the endotracheal tube was less frequent in tracheostomized patients (56.4%) compared with intubated patients (87.1%), while the median duration of invasive mechanical ventilation was longer in the tracheostomy group (17 days; IQR: 12.8–21.8) than in the intubated group (6 days; IQR: 3–9). Among patients who developed VAP, the median age was similar between groups (65 years in tracheostomized vs. 66 years in intubated patients), with a predominance of males (57.7% vs. 64.9%) and high mortality rates in both groups (46.5% vs. 51.4%). No significant association was observed between baseline comorbidities and the risk of VAP, including diabetes mellitus, hypertension, and other conditions not requiring active medical treatment (e.g., history of syphilis, hepatitis B infection, and arrhythmia).

### 3.4. Prior Antibiotic Exposure and VAP Rates

Tracheostomized patients showed lower VAP rates compared with intubated patients when stratified by antibiotic class (14 vs. 23.7 cases per 1000 ventilator-days). In tracheostomized patients, cephalosporins were associated with the lowest VAP rate, followed by quinolones and carbapenems. In intubated patients, the lowest VAP rate was observed with carbapenems, followed by quinolones and cephalosporins (Table 2). Among patients who developed VAP, those treated with carbapenems had lower mortality compared with those receiving other antibiotics (42.4% vs. 57.6%; *p* = 0.04).

### 3.5. Microbiological Findings

Enterobacteriaceae (*Escherichia coli*, *Klebsiella* spp.) and *Pseudomonas* spp. isolates from tracheostomized and intubated patients were analyzed, revealing significant differences in antimicrobial susceptibility profiles (Table 3). Of the 118 specimens submitted for bacteriological analysis, 71 (60.2%) tested positive. Of these, 55.1% were Gram-negative microorganisms, with *Klebsiella pneumoniae* being the most frequent pathogen (15.3%), followed by *Pseudomonas aeruginosa* (13.6%) and *Escherichia coli* (9.3%). In isolates of *E. coli*, a high resistance to ampicillin was evident, with a predominance of intermediate and resistant profiles in both groups (tracheostomized: 85.7%; intubated: 75%). Likewise, a marked resistance to fluoroquinolones, particularly ciprofloxacin, was observed in tracheostomized patients (85.7% resistant), in contrast to the lower resistance in intubated patients (25%). Third-generation cephalosporins (cefotaxime and ceftriaxone) showed resistance rates exceeding 50%, suggesting the presence of extended-spectrum β-lactamase (ESBL)-producing strains. *Klebsiella* spp. exhibited a similar pattern, with high resistance to cephalosporins (up to 60% to ceftriaxone in intubated patients), while carbapenems (ertapenem, imipenem, meropenem) maintained high susceptibility rates (>90%). Amikacin activity was consistently elevated in both groups. In *Pseudomonas* spp., high resistance to cephalosporins was observed, especially ceftriaxone (100%, intrinsic resistance), and there was variable resistance to ceftazidime (66.7% in tracheostomized patients vs. 42.8% in intubated patients). Aminoglycosides and carbapenems showed better activity, although with evidence of emerging resistance. Overall, tracheostomized patients presented a higher proportion of Gram-negative antimicrobial resistance, particularly to fluoroquinolones and *β*-lactams. Resistance to ciprofloxacin in *Escherichia coli* isolates was significantly higher in the tracheostomy group (85.7% vs. 25%; OR = 18.0; 95% CI: 1.2–270; *p* < 0.05). For other antibiotics, such as ceftazidime in *Pseudomonas* spp., a trend toward greater resistance was identified in tracheostomized patients (66.7% vs. 42.8%), although this did not reach statistical significance.

### 3.6. Antimicrobial Resistance Patterns and Profiles

Antimicrobial resistance was assessed using class-based resistance patterns, multidrug resistance (MDR), the multiple antibiotic resistance (MAR) index, and microorganism-specific susceptibility. MDR was primarily observed in *Escherichia coli* isolates, with resistance to cephalosporins and fluoroquinolones consistent in both groups, occurring more frequently among tracheostomized patients. *Klebsiella* spp. primarily showed resistance to cephalosporins without a clear MDR pattern, while data for *Pseudomonas* spp. were insufficient for MDR classification (Supplementary Figure S1).

The MAR index was higher in tracheostomized patients than in intubated patients (0.50 vs. 0.25), indicating a greater overall resistance burden, driven mainly by resistance to fluoroquinolones and cephalosporins (Supplementary Figure S2).

*Staphylococcus aureus* susceptibility profiles were comparable between tracheostomized and intubated patients, with full susceptibility to daptomycin, linezolid, tigecycline, and vancomycin and variable resistance to erythromycin, levofloxacin, moxifloxacin, and tetracycline (Supplementary Figure S3). Overall, antimicrobial resistance was more frequent in tracheostomized patients, particularly due to MDR *E. coli* and increased resistance to key antibiotic classes.

### 3.7. Factors Associated with VAP

A total of 218 patients were included in the analysis, of whom 71 developed VAP and 147 did not (Table 4). In the univariate analysis, the use of broad-spectrum antibiotics was associated with VAP. This association remained significant in the multivariable model (adjusted RR 3.5, 95% CI 1.5–7.5, *p* = 0.005). Mechanical ventilation ≥ 5 days was associated with VAP (OR 6.3, 95% CI 2.6–15.5, *p* < 0.001) and remained significant in the multivariable model (adjusted RR 3.5, 95% CI 1.4–8.7, *p* = 0.007). Chronic pulmonary disease was associated with VAP (OR 11.8, 95% CI 3.3–42.6, *p* < 0.001) and remained significant in the multivariable model (adjusted RR 3.5, 95% CI 1.7–7.3, *p* < 0.001). ICU stay was associated with VAP (OR 3.8, 95% CI 2.0–7.2, *p* < 0.001) and remained significant in the multivariable model (adjusted RR 2.4, 95% CI 1.3–4.2, *p* = 0.003). Supine position (OR 5.3, 95% CI 1.6–18.2, *p* = 0.003), prolonged hospital stay (OR 21.9, 95% CI 2.9–163.3, *p* < 0.001), and tracheostomy (OR 5.5, 95% CI 2.9–10.6, *p* < 0.001) were associated with VAP in the univariate analysis but were not retained in the multivariable model. Antacid use, nasogastric/orogastric tube, emergency intubation, bronchoscopy, enteral feeding, age ≥ 65 years, sedation, and comorbidities were not associated with VAP. Results of the univariable analysis are presented in Supplementary Table S1.

Model comparison using Akaike (AIC) and Schwarz Bayesian (BIC) information criteria demonstrated a consistent improvement in model fit with progressive simplification of the generalized linear models (Supplementary Table S2). The full model, including all candidate variables, showed the highest AIC (206.2) and BIC (236.7) values, indicating the poorest fit among the evaluated models despite maximal adjustment. Sequential removal of variables resulted in a marked reduction in AIC and BIC values, supporting improved model parsimony without compromising statistical significance. Notably, exclusion of demographic confounders and subsequently the prognostic variable led to substantial gains in model performance. The most parsimonious model, comprising broad-spectrum antibiotics, mechanical ventilation ≥ 5 days, chronic pulmonary disease, and ICU stay, achieved the lowest AIC and BIC values, indicating the best overall fit. Importantly, this model retained strong statistical significance (*p* < 1.3 × 10^−14^) and included clinically plausible determinants of VAP.

## 4. Discussion

This study provides real-world evidence on the relationship between tracheostomy and VAP in a retrospective matched cohort of mechanically ventilated ICU patients. Several important findings emerged. First, tracheostomized patients exhibited a substantially higher cumulative incidence of VAP than patients managed exclusively with endotracheal intubation. Second, incidence density rates were nearly identical between groups when ventilator exposure time was considered. Third, tracheostomy was not retained as an independent factor associated with VAP after multivariable adjustment. Collectively, these findings suggest that the higher crude occurrence of VAP among tracheostomized patients is largely explained by prolonged exposure to mechanical ventilation and ICU care rather than by the procedure itself.

Consistent with previous literature [19,20,21], our multivariable analysis supports a multifactorial model of VAP risk. Broad-spectrum antibiotic exposure, mechanical ventilation lasting ≥5 days, chronic pulmonary disease, and prolonged ICU stay remained independently associated with VAP, whereas tracheostomy did not. These findings reinforce the concept that tracheostomy is more likely a marker of prolonged critical illness and healthcare exposure than an independent causal determinant of infection. The robustness of these predictors was supported by model-selection procedures showing optimal performance of the most parsimonious model according to AIC and BIC criteria.

The association between broad-spectrum antibiotic use and VAP deserves particular attention. Although antimicrobial therapy is frequently necessary in critically ill patients, prolonged or inappropriate exposure may alter respiratory microbiota, select resistant organisms, and promote colonization by opportunistic pathogens [8,19]. In our study, stratified analyses showed lower VAP rates among tracheostomized patients across antibiotic classes despite their higher cumulative incidence, emphasizing the complex interaction between antimicrobial therapy, exposure duration, and infection risk. These findings further highlight the importance of antimicrobial stewardship programs aimed at optimizing antibiotic selection, duration, and de-escalation strategies whenever clinically feasible.

The microbiological findings of our study were broadly consistent with contemporary epidemiological reports of VAP [22,23]. Gram-negative pathogens predominated, accounting for more than half of all isolates, with *Klebsiella pneumoniae*, *Pseudomonas aeruginosa*, and *Escherichia coli* representing the most frequently recovered organisms. Resistance patterns were characterized by reduced susceptibility to fluoroquinolones and third-generation cephalosporins, while carbapenems retained high in vitro activity against most Gram-negative isolates. Although confirmatory testing for extended-spectrum β-lactamase production was not performed, the resistance profiles observed among *E. coli* and *Klebsiella* spp. are compatible with ESBL-producing strains.

Of particular interest was the higher burden of antimicrobial resistance observed among tracheostomized patients. *E. coli* isolates demonstrated markedly higher resistance to ciprofloxacin and were primarily responsible for the multidrug-resistant phenotype identified in our cohort. The higher MAR index observed among tracheostomized patients suggests that prolonged ICU stay, cumulative antibiotic exposure, and selective pressure associated with invasive airway management may contribute to the emergence and persistence of resistant microorganisms [24,25,26]. Although the number of *Pseudomonas* spp. isolates was insufficient for robust MDR classification, its clinical relevance remains substantial because of its intrinsic resistance mechanisms and established association with VAP and adverse outcomes [27]. Likewise, the preserved susceptibility of *Staphylococcus aureus* to vancomycin and linezolid indicates that first-line therapies against Gram-positive pathogens remain effective in our setting [28,29].

From a clinical perspective, our findings refine the interpretation of tracheostomy in relation to VAP risk. Once exposure duration and major confounding factors are considered, tracheostomy does not appear to independently increase susceptibility to VAP. Rather, tracheostomy appears to be a marker of prolonged mechanical ventilation and critical illness rather than an independent determinant of VAP occurrence. This interpretation helps explain why previous studies have reported conflicting results and supports current recommendations favoring individualized decision-making regarding tracheostomy timing [30]. Decisions should therefore be based primarily on the anticipated duration of mechanical ventilation, airway management requirements, and weaning considerations rather than concerns about VAP risk alone.

Overall, the present study contributes to the growing body of evidence indicating that the relationship between tracheostomy and VAP is strongly influenced by temporal dynamics and methodological considerations. The higher cumulative incidence observed among tracheostomized patients appears largely attributable to prolonged exposure to mechanical ventilation and ICU care rather than to the procedure itself. Future studies incorporating time-varying exposure models, competing-risk analyses, and standardized definitions of tracheostomy timing are needed to better characterize the complex interaction between airway management strategies and ventilator-associated complications in critically ill patients.

### Limitations

This study has several limitations that should be considered when interpreting the results. First, tracheostomy was analyzed as a fixed exposure despite occurring after ICU admission. Because exposure duration differed substantially between groups, residual confounding related to the timing of tracheostomy cannot be excluded. In addition, competing clinical outcomes such as death, extubation, and ICU discharge were not formally modeled using competing-risk methods. Second, the retrospective observational design limits causal inference and residual confounding cannot be excluded despite multivariable adjustment. Disease severity scores were not consistently available and therefore could not be incorporated into the analyses. Furthermore, broad-spectrum antibiotic use may partially reflect underlying disease severity rather than a direct causal effect on VAP risk. Third, tracheostomy timing and indication were determined according to routine clinical practice, introducing the potential for indication bias. Fourth, important factors associated with VAP risk, including adherence to preventive ventilator-care bundles, oral hygiene, prior antibiotic exposure, and respiratory tract colonization, were not available. Finally, this was a single-center study, which may limit the generalizability of the findings. Nevertheless, the consistency observed across cumulative incidence analyses, incidence density estimates, and multivariate modeling supports the internal validity of the main findings. In particular, the disappearance of the crude association between tracheostomy and VAP after adjustment suggests that prolonged mechanical ventilation and ICU exposure are likely more important determinants of VAP risk than tracheostomy itself.

## 5. Conclusions

Tracheostomized patients exhibited a higher cumulative incidence of VAP than patients managed exclusively with endotracheal intubation, whereas incidence density rates were similar between groups. However, tracheostomy was not retained as an independent predictor of VAP after multivariable adjustment, suggesting that the observed crude association is largely explained by factors associated with prolonged critical illness and healthcare exposure. Variables associated with VAP occurrence in the exploratory multivariable model included broad-spectrum antibiotic exposure, prolonged mechanical ventilation, chronic pulmonary disease, and longer ICU stay. Given the uncertainty regarding the temporal relationship between some covariates and VAP onset, these findings should be interpreted as exploratory associations rather than causal determinants. These observations provide hypotheses for future studies evaluating the complex interplay between antimicrobial exposure, duration of ventilatory support, and VAP occurrence. Future studies incorporating time-varying exposure models and competing-risk analyses are needed to clarify the relationship between tracheostomy timing and VAP occurrence.

## Figures and Tables

**Table 1:** Patients’ baseline characteristics.

	Overall, *n* = 218	Tracheostomy Group *n* = 55	Endotracheal Intubation Group *n* = 163	*p*-Value
Age, years	67 (50–75)	67 (57–75)	67 (44–74)	N.S.
Gender				N.S.
Male	124 (56.9)	29 (52.7)	95 (58.3)	
Female	94 (43.1)	26 (47.3)	68 (41.7)	
Number of underlying medical conditions *				0.008
0	43 (19.7)	4 (7.3)	39 (23.9)	
1	51 (23.4)	13 (23.6)	38 (23.3)	
2	124 (56.9)	38 (69.1)	86 (52.8)	
Ventilation length, days	9 (4–14)	18 (14–24)	6 (4–10)	0.001
Re-intubation	45 (20.6)	24 (43.6)	21 (12.9)	0.001
VAP	71 (32.6)	34 (61.8)	37 (22.7)	0.001
Length of ICU stay, days	9 (4–15)	17 (12–22.5)	6 (4–10)	0.001
Length of hospital stay, days	19 (12–29.5)	32 (24–39)	16 (10–24)	0.001
ICU mortality	118 (54.1)	29 (52.7)	89 (54.6)	N.S.

Data are presented as mean (±SD), number (%) or median (IQR), N.S.: no significant, VAP: ventilator-associated pneumonia, ICU: intensive care unit. * Underlying comorbidities include type 2 diabetes mellitus, systemic arterial hypertension, chronic obstructive pulmonary disease, immunological diseases, and oncological diseases.

**Table 2:** Antibiotic use and ventilator-associated pneumonia (VAP) rates according to airway management strategy.

Airway Management	Antibiotic Class *	VAP Rate (per 1000 Ventilation Days)
Tracheostomized patients	Carbapenems	50.5
	Cephalosporins	46.2
	Quinolones	48.2
Intubated patients	Carbapenems	100.3
	Cephalosporins	123.6
	Quinolones	101.7

* Treatment methods for serious infections included carbapenems (meropenem, ertapenem and imipenem/cilastatin), cephalosporins (ceftriaxone, ceftazidime, cefotaxime and cefepime), and quinolones (ciprofloxacin and levofloxacin). Rates represent subgroup-specific VAP incidence densities according to prior antibiotic exposure.

**Table 3:** Antimicrobial susceptibility of predominant isolates according to airway management (MIC-based classification).

Ampicillin (S < 8|I = 16|R > 32 µg/mL)										
Microorganism	Tracheostomized (*n*)	S	I	R	R (%)	Intubated (*n*)	S	I	R	R (%)
*Escherichia coli*	7	1	4	2	28.6	4	1	2	1	25.0
*Klebsiella* spp.	3	0	2	1	33.3	8	2	5	1	12.5
*Pseudomonas* spp.	-	-	-	-	-	-	-	-	-	-
Ampicillin/Sulbactam (S < 8/4|I = 16/8|R > 32/16 µg/mL)										
Microorganism	Tracheostomized (*n*)	S	I	R	R (%)	Intubated (*n*)	S	I	R	R (%)
*Escherichia coli*	7	4	1	2	28.6	4	2	2	0	0
*Klebsiella* spp.	3	1	2	0	0	8	2	2	0	0
*Pseudomonas* spp.	-	-	-	-	-	-	-	-	-	-
Amikacin (S < 16|I = 32|R > 64 µg/mL)										
Microorganism	Tracheostomized (*n*)	S	I	R	R (%)	Intubated (*n*)	S	I	R	R (%)
*Escherichia coli*	7	6	1	0	0	4	3	1	0	0
*Klebsiella* spp.	3	3	0	0	0	9	6	3	0	0
*Pseudomonas* spp.	4	3	1	0	0	4	4	0	0	0
Ceftazidime (S < 4|I = 8|R > 16 µg/mL)										
Microorganism	Tracheostomized (*n*)	S	I	R	R (%)	Intubated (*n*)	S	I	R	R (%)
*Escherichia coli*	7	3	1	3	42.9	3	1	0	2	66.7
*Klebsiella* spp.	3	1	0	2	66.7	12	9	1	2	16.7
*Pseudomonas* spp.	6	2	0	4	66.7	7	4	0	3	42.9
Ceftriaxone (S < 1|I = 2|R > 4 µg/mL)										
Microorganism	Tracheostomized (*n*)	S	I	R	R (%)	Intubated (*n*)	S	I	R	R (%)
*Escherichia coli*	7	3	0	4	57.1	4	0	0	4	100
*Klebsiella* spp.	3	1	0	2	66.7	12	7	0	5	41.7
*Pseudomonas* spp.	4	0	0	4	100	7	0	0	7	100
Cefepime (S < 2|I = 4–8|R > 16 µg/mL)										
Microorganism	Tracheostomized (*n*)	S	I	R	R (%)	Intubated (*n*)	S	I	R	R (%)
*Escherichia coli*	7	2	2	3	42.9	4	2	0	2	50
*Klebsiella* spp.	3	1	0	2	66.7	12	7	3	2	16.7
*Pseudomonas* spp.	6	2	0	4	66.7	7	4	0	3	42.9
Ciprofloxacin (S < 0.25|I = 0.5|R > 1 µg/mL)										
Microorganism	Tracheostomized (*n*)	S	I	R	R (%)	Intubated (*n*)	S	I	R	R (%)
*Escherichia coli*	7	0	1	6	85.7	4	2	1	1	25
*Klebsiella* spp.	3	1	0	2	66.7	12	8	2	2	16.7
*Pseudomonas* spp.	6	3	2	1	16.7	7	5	0	2	28.6

Data are expressed as number of isolates. Susceptibility interpreted according to CLSI guidelines. ESBLs: extended-spectrum β-lactamases. Intrinsic resistance in *Klebsiella* spp. and *Pseudomonas* spp. is not reported (-). R (%): percentage of resistant isolates.

**Table 4:** Variables independently associated with VAP occurrence in the exploratory multivariable model during ICU stay.

Exploratory Variable	Adjusted RR	95% CI	*p*-Value
Broad-spectrum antibiotic use	3.5	1.5–7.5	0.005
Mechanical ventilation ≥ 5 days	3.5	1.4–8.7	0.007
Chronic pulmonary disease	3.5	1.7–7.3	0.001
ICU stay	2.4	1.3–4.2	0.003

VAP: ventilator-associated pneumonia. ICU: intensive care unit. Adjusted effect estimates were estimated using a generalized linear model with binomial distribution and complementary log–log link function, adjusted for age and sex. Because the temporal relationship between some covariates and VAP onset could not be established with certainty, these estimates should be interpreted as exploratory associations rather than causal predictors. Variables not retained in the final model are presented in Supplementary Table S1.

## Data Availability

The datasets generated and analyzed during the present study are not publicly available but can be made available from the corresponding authors on reasonable request.

## Supplementary Materials

The following supporting information can be downloaded at https://www.mdpi.com/article/10.3390/jcm15124811/s1. Figure S1: Class-based multidrug resistance pattern in *Escherichia coli*; Figure S2: Mean antimicrobial resistance across tested antibiotics in tracheostomized versus intubated patients; Figure S3: Comparative antimicrobial susceptibility profiles of Gram-positive isolates in tracheostomized versus intubate patients; Table S1: Variables evaluated but not retained in the final multivariable model; Table S2: Bayesian (BIC) and Akaike (AIC) Information Criteria for generalized linear models explaining ventilator-associated pneumonia adjusted by age and gender.

## Abbreviations

aRRs	Adjusted relative risk
AIC	Akaike Information Criterion
AST	Antimicrobial susceptibility testing
ARDS	Acute respiratory distress syndrome
BIC	Bayesian Information Criterion
BMI	Body mass index
HAIs	Healthcare-associated infections
HAP	Hospital-acquired pneumonia
ICU	Intensive care unit
INICC	International Nosocomial Infection Control Consortium
MAR	Multiple antibiotic resistance
MDR	Multidrug-resistant
MIC	Minimum inhibitory concentration
PMV	Prolonged mechanical ventilation
STROBE	Strengthening the Reporting of Observational Studies in Epidemiology
VAP	Ventilator-associated pneumonia
