# Supplementary material for: Tracheostomy and Ventilator-Associated Pneumonia in Mechanically Ventilated ICU Patients: A Retrospective Matched Cohort Study
Source: J Clin Med. 2026 Jun 21;15(12):4811. doi: 10.3390/jcm15124811 (PMC13301646; doi:10.3390/jcm15124811)
Supplement: Supplementary file 1 [file jcm-15-04811-s001.zip › Supplementary Figure S1_Dra.pdf]

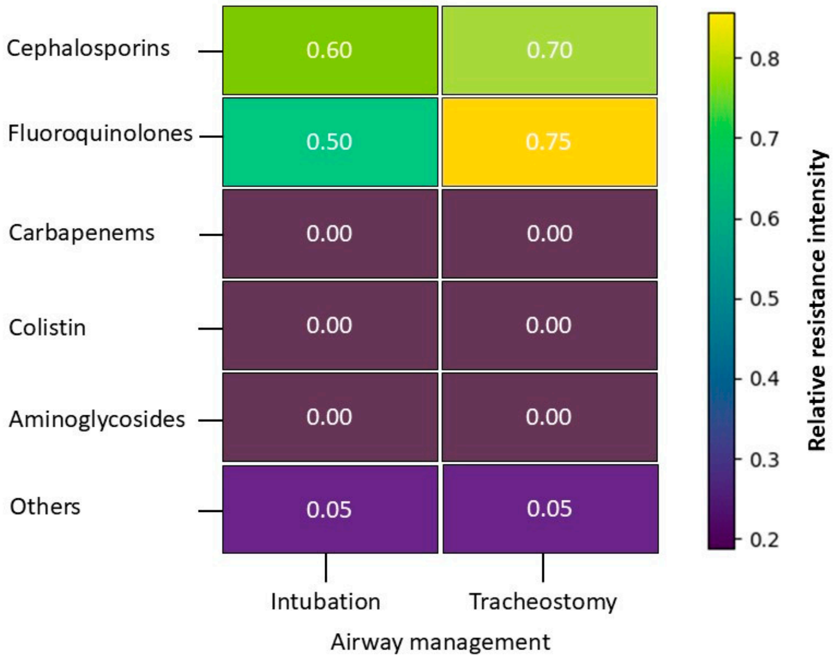

Supplementary Figure S1. Class–based multidrug resistance pattern in *Escherichia coli*. Heatmap illustrating the class–based resistance profile of *E. coli* across study groups. Color intensity reflects the relative magnitude of resistance. A consistent pattern of co–resistance to cephalosporins and fluoroquinolones is observed, indicating that multidrug resistance (MDR) is predominantly driven by *E. coli*. In contrast, carbapenems, colistin, aminoglycosides, and others pharmacology agents show preserved susceptibility across both groups.
