# Supplementary material for: Tracheostomy and Ventilator-Associated Pneumonia in Mechanically Ventilated ICU Patients: A Retrospective Matched Cohort Study
Source: J Clin Med. 2026 Jun 21;15(12):4811. doi: 10.3390/jcm15124811 (PMC13301646; doi:10.3390/jcm15124811)
Supplement: Supplementary file 1 [file jcm-15-04811-s001.zip › Supplementary Figure S2.pdf]

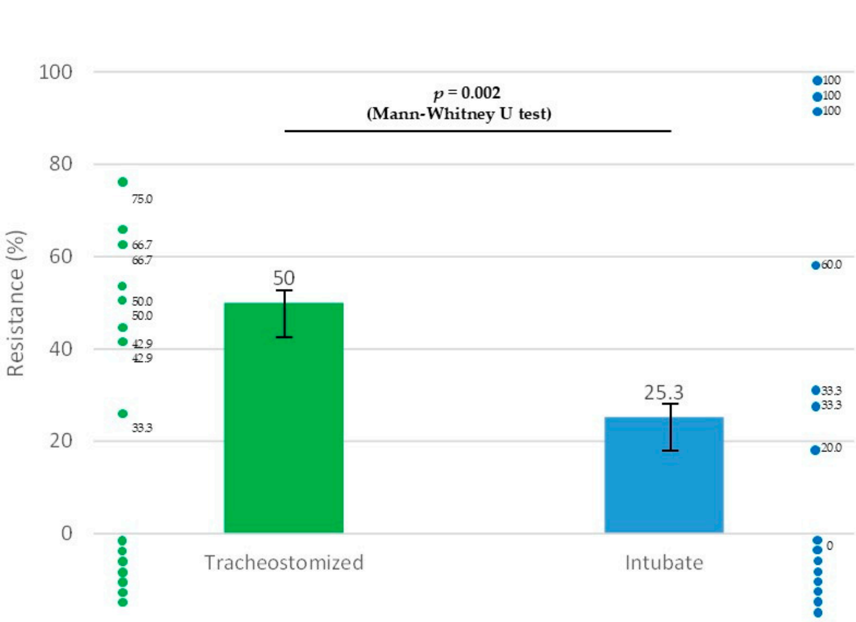

Antibiotic	Tracheostomized patients R %	Intubate patients R %
1. Levofloxacin	75.0	100
2. Norfloxacin	66.7	100
3. Cefazolin	66.7	60.0
4. Cefotaxime	50.0	100
5. Cephalothin	42.9	33.3
6. Cefuroxime	42.9	33.3
7. Fosfomycin	33.3	20.0
8. Amoxicillin/clavulanic acid	0	0
9. Colistin	0	0
10. Doripenem	0	0
11. Nitrofurantoin	0	0
12. Tetracycline	0	0
13. Tigecycline	0	0
14. TMP/SMX	0	0
15. Tobramycin	0	0

Supplementary Figure S2. Mean antimicrobial resistance across tested antibiotics in tracheostomized versus intubate patients. Bars represent the mean proportion of resistant (R%) across all evaluated antibiotics, while dots indicate the resistance percentage for each individual antibiotic. Tracheostomized patients showed a higher overall resistance burden compared to intubate patients, mainly driven by cephalosporins and fluoroquinolones. Statistical comparison was performed using the Mann–Whitney U test.
