# Supplementary material for: Tracheostomy and Ventilator-Associated Pneumonia in Mechanically Ventilated ICU Patients: A Retrospective Matched Cohort Study
Source: J Clin Med. 2026 Jun 21;15(12):4811. doi: 10.3390/jcm15124811 (PMC13301646; doi:10.3390/jcm15124811)
Supplement: Supplementary file 1 [file jcm-15-04811-s001.zip › Supplementary Figure S3_Dra.pdf]

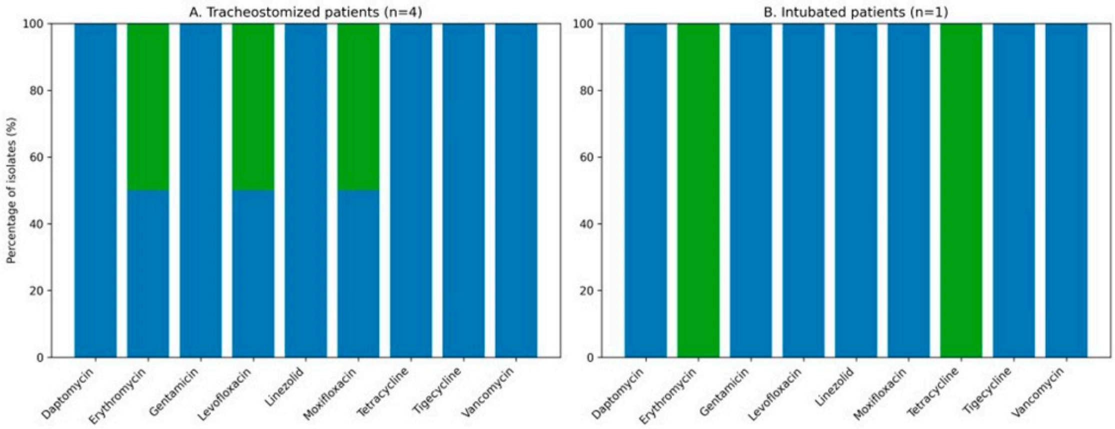

Supplementary Figure S3. Comparative antimicrobial susceptibility profiles of Gram-positive isolates in tracheostomized versus intubated patients. Stacked bar charts showing the percentage distribution of susceptible and resistant Gram-positive bacterial isolates to selected antimicrobial agents in tracheostomized (**A**) and intubated (**B**) patients. Tracheostomized patients exhibited higher resistance rates to macrolides and fluoroquinolones, whereas isolates from intubated patients remained broadly susceptible to most tested antibiotics. These patterns may reflect differences in cumulative antibiotic exposure and duration of intensive care support.
