# Supplementary material for: Tracheostomy and Ventilator-Associated Pneumonia in Mechanically Ventilated ICU Patients: A Retrospective Matched Cohort Study
Source: J Clin Med. 2026 Jun 21;15(12):4811. doi: 10.3390/jcm15124811 (PMC13301646; doi:10.3390/jcm15124811)
Supplement: Supplementary file 1 [file jcm-15-04811-s001.zip › Supplementary Table S1.pdf]

Supplementary Table S1. Variables evaluated but not retained in the final multivariable model.

Variable	VAP (*n*)	No VAP (*n*)	Univariate Analysis * (OR)	95% CI	*p*-Value
Antacid use					
Yes	41	76	–	–	N.S.
No	30	71			
Supine position					
Yes	68	119	5.3	1.6–18.2	0.003
No	3	28	(reference)		
Nasogastric/orogastric tube					
Yes	68	138	–	–	N.S.
No	3	9			
Emergency intubation					
Yes	70	146	–	–	N.S.
No	1	1			
Bronchoscopy					
Yes	1	0	–	–	N.S.
No	70	147			
Enteral nutrition					
Yes	66	130	–	–	N.S.
No	4	13			
Age > 65 years					
Yes	36	82	–	–	N.S.
No	35	65			
Sedation					
Yes	69	135	–	–	N.S.
No	2	12			
Baseline comorbidities					
Yes	59	117	–	–	N.S.
No	12	30			
Tracheostomy					
Yes	34	21	5.5	2.9–10.6	0.001
No	37	126	(reference)		

Data are presented as number of patients. Univariate associations with ventilator-associated pneumonia were assessed using odds ratios (ORs) with 95% confidence intervals (95% CI). Variables that did not demonstrate a statistically significant association in univariate analysis or did not improve model performance were excluded from the final multivariable model. "N.S." indicates non-significant. Reference categories are indicated where applicable. OR, odds ratio; CI, confidence interval; VAP, ventilator-associated pneumonia.
