# Supplementary material for: Tracheostomy and Ventilator-Associated Pneumonia in Mechanically Ventilated ICU Patients: A Retrospective Matched Cohort Study
Source: J Clin Med. 2026 Jun 21;15(12):4811. doi: 10.3390/jcm15124811 (PMC13301646; doi:10.3390/jcm15124811)
Supplement: Supplementary file 1 [file jcm-15-04811-s001.zip › Supplementary Table S2.pdf]

Supplementary Table S2. Bayesian (BIC) and Akaike (AIC) Information Criteria for generalized linear models explaining ventilator–associated pneumonia adjusted by age and gender.

Generalized Linear Models	BIC	AIC	*p*–value
Tracheostomy + ABS + Ventilation + Comorbidities + ICU admission + Mortality + Age + Gender	236.7	206.2	$<4.9 \times 10^{-13}$
Tracheostomy + ABS + Ventilation + Comorbidities + ICU admission + Mortality + Age	221.8	194.7	$<1.5 \times 10^{-5}$
Tracheostomy + ABS + Ventilation + Comorbidities + ICU admission + Mortality	96.4	72.8	$<4.6 \times 10^{-14}$
Tracheostomy + ABS + Ventilation + Comorbidities + ICU admission	73.4	53.1	$<1.8 \times 10^{-14}$
ABS + Ventilation + Comorbidities + ICU admission	58.1	41.2	$<1.3 \times 10^{-14}$

Abbreviations and notes

BIC: Bayesian Information Criterion (Schwarz criterion)

AIC: Akaike Information Criterion

Note: Lower AIC and BIC values indicate better model fit.

Putative factors

Tracheostomy: Surgical tracheostomy procedure

ABS: Broad-spectrum antibiotics

Ventilation: Mechanical ventilation >5 days

Comorbidities: History of chronic pulmonary disease

ICU admission: Admission to intensive care unit

Prognostic factor

Mortality: Death of patient

Confounding factors

Age: Chronological age

Gender: Female/Male.
